# 

**Published:** 1915-04

**Authors:** 


### Deaths.

Dr. E. H. Scharnberg of Coupland died in Brenham, March 3.

Dr. E. J. Owens, a well known physician, died in Caddo Mills, March 22.

Dr. R. J. Swain, who had practiced medicine at Pittsburg, Texas, for the past thirty-six years, passed away in that city March 11.

Because of ill health Dr. J. B. Berry of Detroit, Texas, took his own life in that city March 10. The doctor was about 50 years old.

Mrs. Elizabeth Aston, wife of Dr. C. C. Aston, died in Dallas, March 10, and was taken to Farmersville for burial.

